# Brief review of the chicken Major Histocompatibility Complex: the genes, their distribution on chromosome 16, and their contributions to disease resistance

**DOI:** 10.3382/ps/pev379

**Published:** 2016-06-17

**Authors:** Marcia M. Miller, Robert L. Taylor

**Affiliations:** * Beckman Research Institute, City of Hope, Department of Molecular and Cellular Biology, Duarte, CA 91010; † Division of Animal and Nutritional Sciences, West Virginia University, Morgantown, WV 26506

**Keywords:** Chicken, GGA 16, MHC, gene map, genetics of resistance to infectious disease

## Abstract

Nearly all genes presently mapped to chicken chromosome 16 (**GGA 16**) have either a demonstrated role in immune responses or are considered to serve in immunity by reason of sequence homology with immune system genes defined in other species. The genes are best described in regional units. Among these, the best known is the polymorphic major histocompatibility complex-*B* (**MHC-*B***) region containing genes for classical peptide antigen presentation. Nearby MHC-*B* is a small region containing two *CD1* genes, which encode molecules known to bind lipid antigens and which will likely be found in chickens to present lipids to specialized T cells, as occurs with CD1 molecules in other species. Another region is the MHC-*Y* region, separated from MHC-*B* by an intervening region of tandem repeats. Like MHC-*B*, MHC-*Y* is polymorphic. It contains specialized class I and class II genes and c-type lectin-like genes. Yet another region, separated from MHC-*Y* by the single nucleolar organizing region (***NOR***) in the chicken genome, contains olfactory receptor genes and scavenger receptor genes, which are also thought to contribute to immunity. The structure, distribution, linkages and patterns of polymorphism in these regions, suggest GGA 16 evolves as a microchromosome devoted to immune defense. Many GGA 16 genes are polymorphic and polygenic. At the moment most disease associations are at the haplotype level. Roles of individual MHC genes in disease resistance are documented in only a very few instances. Provided suitable experimental stocks persist, the availability of increasingly detailed maps of GGA 16 genes combined with new means for detecting genetic variability will lead to investigations defining the contributions of individual loci and more applications for immunogenetics in breeding healthy poultry.

## INTRODUCTION

The major histocompatibility complex (**MHC**) is a gene region possessed by all higher vertebrate species. Many of the genes within the MHC contribute to immunity. MHC encoded class I and class II molecules are central in defining the specificity of adaptive immune responses. MHC class I and class II molecules provide a context in which pathogens are recognized. Presentation by MHC molecules is essential for developing vaccinal immunity. MHC class I and class II molecules are typically highly polymorphic and polygenic. Other MHC genes, some of which are also polymorphic and polygenic, contribute to immunity in additional ways. The chicken MHC is especially interesting because polymorphic MHC class I and class II genes are localized into two regions (MHC-*B* and MHC-*Y*) on the same chromosome. The MHC-*B* and MHC-*Y* haplotypes assort independently as the result of an intervening region that supports highly frequent recombination. The chicken MHC is of interest since particular MHC-*B* haplotypes and, in a few instances, alleles at particular MHC-*B* loci are known to exert major genetic effects in the incidence of infectious diseases, such as Marek's disease (MD). Genetic lines of chickens selected for different MHC-*B* haplotypes have provided abundant evidence for the major contribution of the MHC to disease resistance. A detailed gene map exists for MHC-*B* in the red Jungle fowl reference genome in which two class I genes, two class IIβ genes and associated genes are located (Shiina et al., 2007). Additional sequence data is now available for the large family of *BG* genes (Salomonsen et al., 2014) and two nearby MHC class I-like genes, the *CD1* genes (Maruoka et al., 2005; Miller et al., 2005; Salomonsen et al., 2014; Salomonsen et al., 2005; Shiina et al., 2007). While not yet fully sequenced, additional sections containing MHC-*Y* genes provide evidence for a number of specialized MHC class I, MHC class II, c-type lectin-like, and linked olfactory receptors (***OR***), and scavenger receptor (***SRCR***) genes (Miller et al., 2014; Rogers et al., 2003). A nomenclature guide provides the naming conventions for MHC-*B* and MHC-*Y* haplotypes and genes (Miller et al., 2004).

Not all investigators agree on where to draw the boundaries between genes within the major histocompatibility complex and those in adjacent regions. This is not surprising since the region is organized in diverse ways in different species. In addition, the original name – major histocompatibility complex – reflects an experimental outcome in a pre-genomic era. Now, with genomics, it is possible to have an overview that reveals far more about the genes within the region. In this review an inclusive definition has been adopted to encompass the different types of genes commonly found in the MHC genomic region. Most of these contribute in some way to immunity. The majority of the genes mapped to GGA 16 either have a demonstrated role typical of the MHC complex or are considered likely to have a role in immunity. The genes mapped in chicken to GGA 16 support consideration of the MHC as a region where a variety of co-functioning and co-evolving genes are concentrated together.

## DISTRIBUTION OF GENES ON CHICKEN CHROMOSOME 16 (GGA 16)

**Figure 1.**
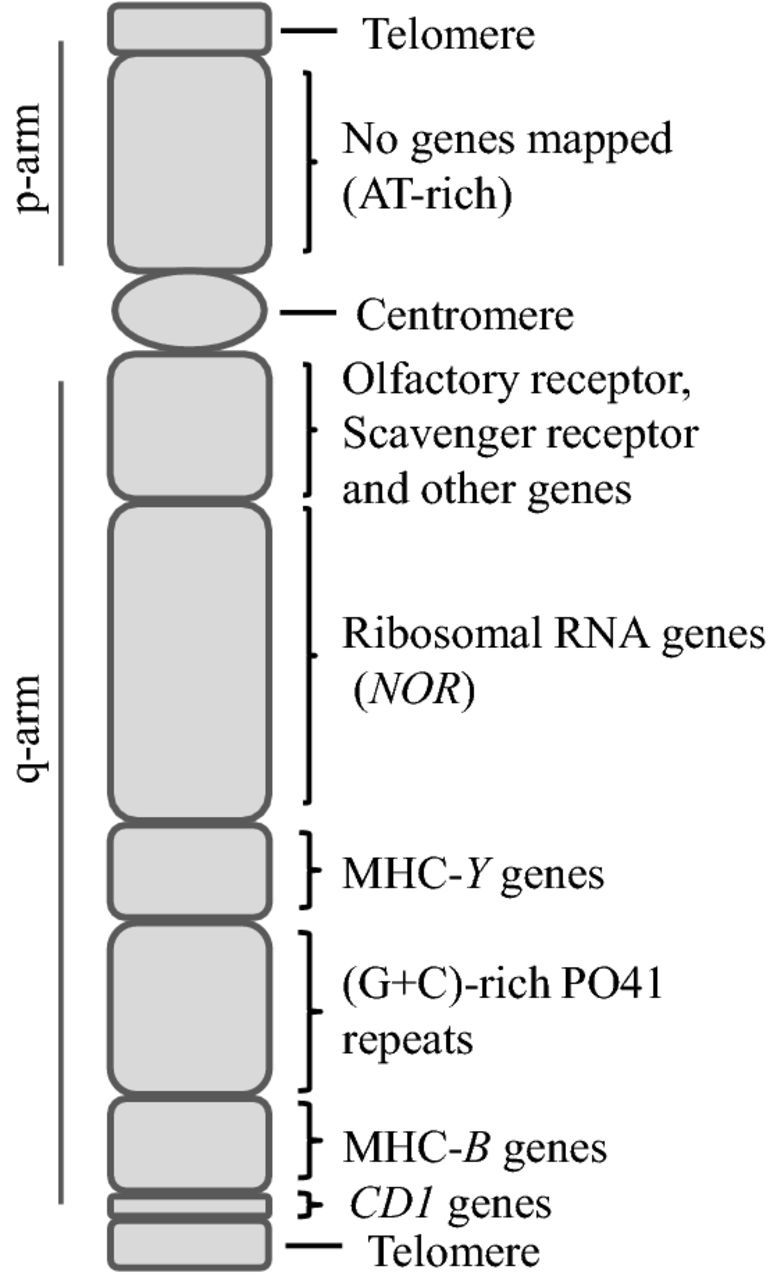
MHC-*B* and MHC-*Y* are located on the q-arm of chicken chromosome 16 (GGA 16) distal to the *NOR* and are separated by PO41 repeats. MHC-*Y* displays linkage with the olfactory receptor and scavenger receptor genes. MHC-*B* haplotypes assort independently from MHC-*Y haplotypes*. Two *CD1* class I genes are located distal to MHC-*B*. Adapted from Miller et al. 2014.

GGA16 is one of the larger microchromosomes in the chicken karyotype. Genes have been mapped to the q-arm with no genes as yet assign to the p-arm (Figure 1) (Delany et al., 2009; Miller et al., 2014). In the current map, the first genes distal to the centromere on GGA 16 are multiple copies of *OR* and cysteine-rich domain *SRCR* genes. These were identified as likely mapping to GGA 16 by trisomy mapping(**TM**)/comparative genomic hybridization (**aCGH**) and then located by FISH (Miller et al., 2014). Distal to the *OR* and *SRCR* genes is the single ***NOR*** (nucleolar organizing region) in the chicken genome. This is the chromosomal region around which the nucleolus forms. The *NOR* occupies a large portion (5–7 Mb) of the q-arm and contains repetitive sets (∼150 copies) of 18S, 5.8S, and 28S ribosomal RNA genes. Located just distal to the *NOR* is the MHC-*Y* region.

Distal to MHC-*Y* is a long array of (G+C)-rich pattern of 41 nucleotides (**PO41**) tandem repeats (Solinhac et al., 2010). The chiasma observed in GGA 16 lampbrush chromosomes occur in the PO41 region suggesting that these tandem repeats are the source of the independent assortment of MHC-*Y* haplotypes from MHC-*B* haplotypes (Solinhac et al., 2010). The MHC-*B* region distal to the PO41 repeats has been studied extensively for its role in antigen presentation and disease resistance. Distal to MHC-*B* are additional genes including two members of the ancient *CD1* gene family encoding specialized antigen presenting molecules.

Additional genes mapping to GGA 16 but not yet located at high resolution include *BLA*, encoding a class IIα chain (Kaufman et al., 1995a; Salomonsen et al., 2003); G9 (BAT8) encoding a methyl transferase mapping to the complement gene region in other species (Spike et al., 1996) and a fB gene, a gene for complement factor B (Kaufman et al., 1999). An MHC class II restriction fragment polymorphism has provided evidence of an additional class II gene that is not located (Juul-Madsen et al., 2000b). Additional candidates identified in the TM/CGH study as candidates for mapping to GGA 16 include members of the immunoglobulin gene superfamily that may also be contributing to immunity (Miller et al., 2014). Further sequencing and cytogenetic mapping studies are needed to confirm these assignments and to locate these genes.

## OLFACTORY AND SCAVENGER RECEPTOR GENES

Eleven chrUn_random RefSeq sequences containing *OR* and *SRCR* genes were assigned to GGA 16 by TM/aCGH and mapped to the proximal region of the GGA 16 q-arm by FISH (Miller et al., 2014). Segregation of *SRCR* gene restriction fragments in analyses of fully pedigreed families has provided evidence that the region is polymorphic and that *OR/SRCR* genes segregate in linkage with MHC-*Y* region genes (Miller et al., 2014). Multiple types of *OR* genes are present in the OR/SRCR RefSeq sequences including member of the *OR14J1* family. Interestingly, *OR14J1* genes are also found in the extended region of the human MHC (Ehlers et al., 2000) so with this finding in the chicken genome there is evidence for a long-term association of *OR14J1* genes with the MHC.

Nine *SRCR* genes were contained in the sequences assigned to GGA 16 in the TM/CGH study (Miller et al., 2014). The genes are easy to identify because they contain multiple sequences for the highly conserved and ancient scavenger receptor cysteine-rich domain (Herzig et al., 2010; Miller et al., 2014). In sequence alignments, the chicken SRCR genes are most similar to *WC1/CD163* genes. Recent data suggest that cattle WC1 molecules are pattern recognition co-receptors that guide responses of γδ T cells to bacteria (Hsu et al., 2015; Telfer and Baldwin, 2015). The scavenger receptor CD163, a “jack-of-all-trades” molecule with restricted expression on the surfaces of monocytes and macrophages, is apparently multifunctional, mediating endocytosis of a variety of ligands (including hemoglobin, bacteria, and viruses), serving as an adhesion receptor (on erythroblasts) and as an immunomodulator operating under immunosuppressive conditions (Van Gorp et al., 2010). The chicken *SRCR* genes are candidates for serving in immunity and fit well within the inclusive definition of the MHC used in this review.

## MHC-*Y* REGION GENES

The MHC-*Y* region, originally named *Rfp*-*Y*, was discovered when chicken MHC-*B* class I and class II gene probes revealed DNA restriction fragments from a fully pedigreed family that were inconsistent with the assigned MHC-*B* haplotypes (Briles et al., 1993). Soon after this discovery, two MHC class I, two MHC class II genes and a c-type lectin-like gene originally assigned to MHC-*B* (Guillemot and Auffray, 1989) were reassigned to MHC-*Y* and localized to GGA 16 (Miller et al., 1994; Miller et al., 1996). Assembly of the full sequence of the MHC-*Y* region is particularly challenging, but work is progressing (Miller, unpublished data). The published sequence data provides evidence for two c-type lectin-like genes, a full class I gene, a truncated class IIβ gene and CR1 repeat within the region (Rogers et al., 2003).

MHC-*Y* haplotypes are assigned on the basis of restriction fragment polymorphisms. Initially, these haplotypes were identified with MHC-*B* probes that cross-hybridize with MHC-*Y* class I and class II gene sequences (Briles et al., 1993; Miller et al., 1994; Miller et al., 1996; Pharr et al., 1997). MHC-*Y* specific probes include probe *18.1*, from an intergenic region in MHC-*Y* (Guillemot and Auffray, 1989; Juul-Madsen et al., 1997) and *163/164f*, a probe from a MHC-*Y*-specific region of the YF1*7.1 gene (Afanassieff et al., 2001).

**Figure 2.**
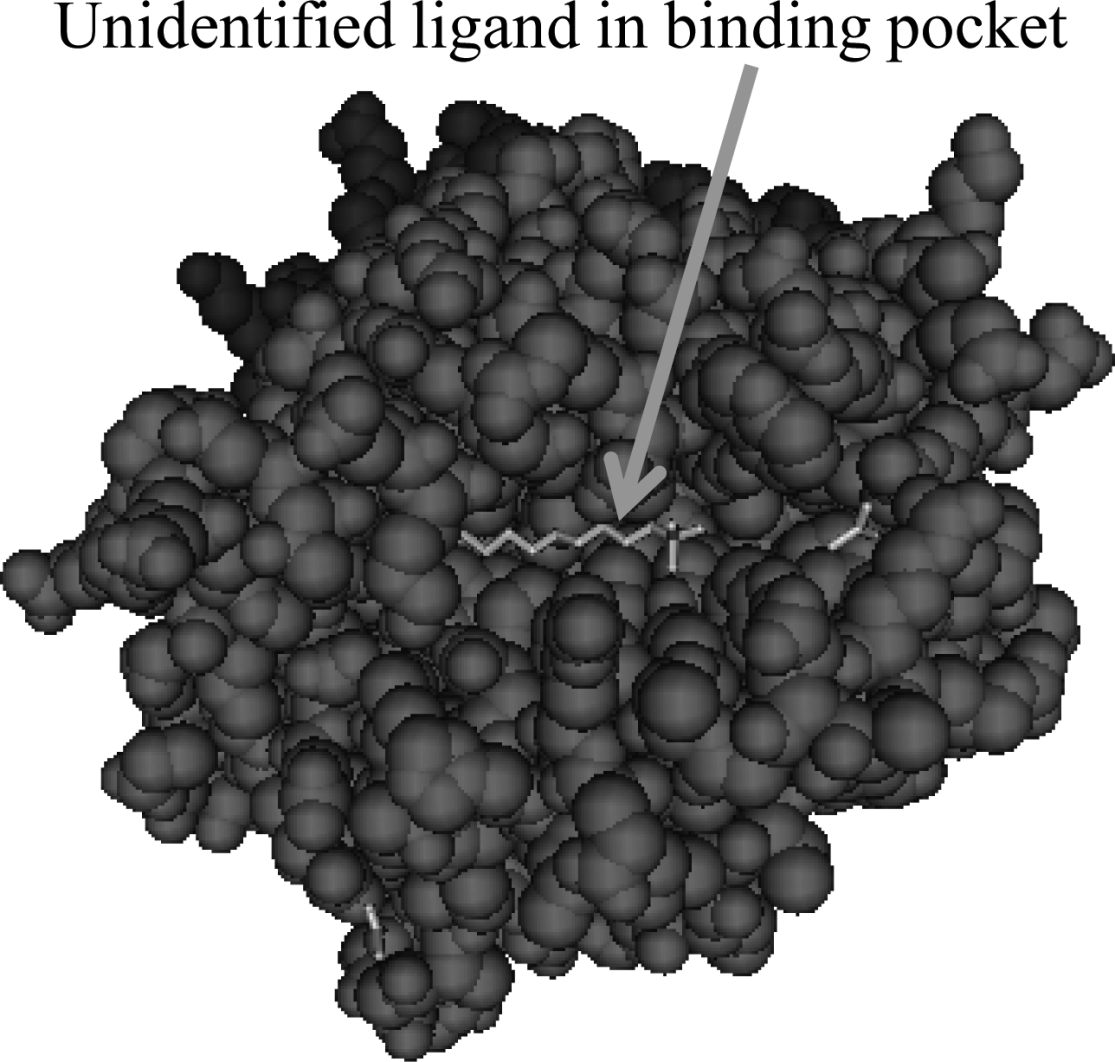
Structure of YF1*7.1 showing surfactant bound within the ligand binding cleft. From NCBI PBD ID: 3P73.

Early data demonstrated that MHC-Y antigens serve as weak alloantigens (Pharr et al., 1996). Similar results were reported in a study of three additional MHC-*Y* haplotypes (Thoraval et al., 2003). Other investigators (Afanassieff et al., 2001; Hunt et al., 2006) working with highly inbred and closed lines have shown that at least one MHC-*Y* class I genes is polymorphic and dynamically expressed. At least one MHC-Y class I (YF) molecule is alloimmunogenic (Hunt et al., 2006). Initial evidence of MHC-Y class I antigen binding groove specialization (Afanassieff et al., 2001; Afanassieff et al. 2000) was confirmed by structural studies (Figure 2) of YF1*7.1 (Hee et al., 2010; Hee et al., 2009) showing non-peptidic ligands logged within its hydrophobic binding groove. The structure of YF1*7.1 defined a new MHC class I paradigm; a molecule with the structure of a classical MHC class I molecule, but binding “non-classical” non-peptide ligands. A more recent structural study has revealed human **MR1** (MHC class I (MHC-I)-like related molecule) as a second member of this group (Kjer-Nielsen et al., 2012). MR1 has a classical structure, but presents B vitamin metabolites of microbial origin. These findings suggest that MHC-Y class I molecules may contribute in guiding immune responses of a specialized nature.

As a note of explanation, YF1*7.1 is named following the recommended chicken MHC nomenclature (Miller et al., 2004). Following standard conventions gene names are italicized. Briefly, “*Y*” designates the gene region. In chickens “*F*” is used to identify a chicken class I gene. The “*1*” is to distinguish the first *F* locus in the haplotype from additional class I loci in the haplotype, *e.g.*, the *YF7* haplotype has two class I loci, *YF1* and *YF2*. An asterisk is used to separate locus from haplotype. “*7.1*” is the haplotype found in Reaseheath Line C and its derivatives. Because MHC-*Y* haplotypes are only partially defined, decimal designations (“*7.1*”, “*7.2*”, and so on) are used to distinguish apparently identical haplotypes originating from different genetic stock.

In early analyses of chicken MHC genes, six *BLβ* genes were described in the Line CB genome (Zoorob et al., 1990; Zoorob et al., 1993). Following MHC-*Y* identification and mapping, three of these genes, originally named *BLβIII*, *BLβIV*, and *BLβV*, were reassigned to MHC-*Y* and renamed *YLB1*, *YLB2* and *YLB3* (Miller et al., 2004; Miller et al., 1994; Miller et al., 1996). These three MHC-*Y* loci share sequence similarities and more limited polymorphism that set them apart from the MHC-*B* class IIβ loci (Zoorob et al., 1993).

The c-type lectin-like genes mapping to MHC-*Y* were identified in the first chicken MHC map as part of MHC-*B* (Bernot et al., 1994). Bernot and colleagues identified a locus (17.5) on cosmid clone cβ17 as a lectin-like gene for which a cDNA clone (17.5.3) was isolated. Southern blots with 17.5.3 provided evidence for a family of polymorphic genes The cβ17 clone was later assigned to MHC-*Y* (Miller et al., 1994). Two additional C-type lectin-like genes are currently mapped to MHC-*Y* (Rogers et al., 2003)*.* Now identified as *Ylec* genes, these genes are similar to the mammalian genes encoding C-type lectin-like receptors that guide mammalian natural killer cell responses (Iizuka et al., 2003).

## THE MHC-*B* REGION GENES

Serological and genetic data reported by Briles in 1950 provided the first evidence for the presence of MHC-*B* as an alloantigen system displayed on the surfaces of red blood cells (Briles et al., 1950). Gilmour's independent discovery nine years later confirmed the presence of this highly polymorphic system (Gilmour, 1959). Schierman and Nordskog (Schierman and Nordskog, 1961) linked the B alloantigen system to the chicken major histocompatibility complex by demonstrating that skin grafts were rejected more rapidly if donor and recipient were not matched for the B system compared with other blood alloantigens. Later, recombinant MHC-*B* haplotypes enabled Pink and colleagues (Pink et al., 1977) to define three classes of MHC-B antigens each with a different tissue distribution indicating the presence of at least three MHC loci. Further work showed that immune cell cooperation was restricted by MHC*-B* antigens (Maccubbin and Schierman, 1986; Vainio et al., 1984; Weinstock et al., 1989).

The first gene map for the *B12* haplotype encompassed multiple class I and class II genes, additional loci and a portion of the *NOR* in four cosmid clone clusters (Guillemot et al., 1989). Following the discovery of MHC-*Y* (Briles et al., 1993; Miller et al., 1988), three of the four clusters, including the one containing *rDNA* genes, were reassigned to MHC-*Y* leaving MHC-*B* represented by a single cluster of genes (Miller et al., 1994). When sequenced, this cosmid revealed 19 genes within a 92 kb region including two class Iα genes, two class IIβ genes and two c-type lectin-like receptor genes (Kaufman et al., 1999). Subsequently, by sequencing *B12* BAC clones, six *TRIM* genes and a guanine nucleotide-binding gene were located nearby (Ruby et al., 2005; Zoorob et al., 1996).

### The Current MHC-B Gene Map

**Figure 3.**
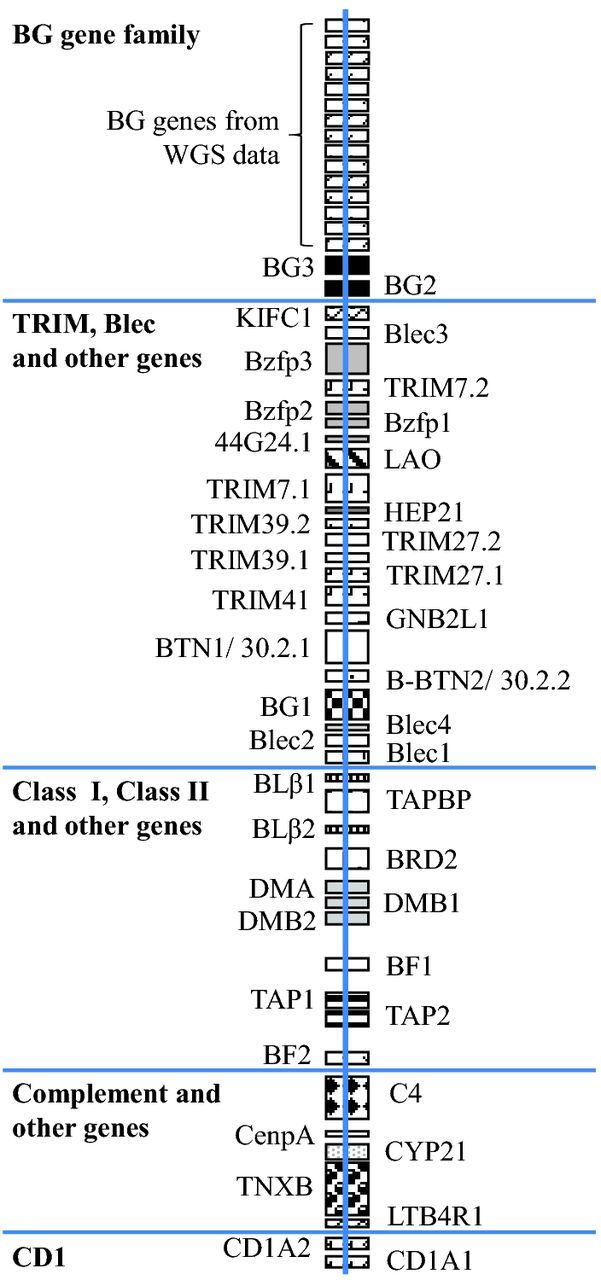
Gene Map for the red Jungle fowl MHC-*B* haplotype. Adapted from Shiina et al. 2007 and Salomonsen et al. 2014.

The foundation for the current Red Junglefowl (**RJF**) MHC-*B* region gene map (Figure 3) is the full sequence determinations made for three overlapping RJF BAC clones encompassing 242 kb (Shiina et al., 2007). The sequence revealed 46 genes within 242 kb. This extended the original MHC-*B* map from made from the *B12* cosmid clone, adding one additional (pseudo) gene within the original map and fully organizing 21 additional genes upstream and five additional genes downstream of the *B12* 92 kb map. The recent publication of the *B12 BG* haplotype (Salomonsen et al., 2014) has made a significant addition to the MHC-*B* map. Twelve *BG* genes, identified in the *B12* haplotype sequence, were found distributed in two tandem repeats upstream of the *TRIM* and *Blec* sub-region. These data were used to tentatively order the *BG* haplotype in the RJF whole genome shotgun (WGS) project. At least 15 RJF *BG* genes (apparently lacking the insert of dissimilar genes that disrupts the continuity of *BG* genes in *B12 BG* haplotype) are thought to be located upstream of *BG3* (noted by a cluster of boxes at the top of Figure 3). The number of *BG* genes apparently differs among MHC-*B* haplotypes providing evidence for gene copy number variation within the chicken MHC.

### BG Gene Family Sub-Region

Almost all the genes within the *BG* family are clustered in tandem repeats in which indels, as well as simpler polymorphisms, distinguish haplotypes (Salomonsen et al., 2014). Sequence comparisons indicate that most *BG* genes are hybrid genes suggesting that recombination contributes to diversity among haplotypes. Only two genes fall outside the repeat cluster. One, *BG0* is located away from the MHC on chromosome GGA 2. The other, *BG1* is positioned more than 115 kb away from the cluster near the MHC class I and class II loci (Kaufman et al., 1999; Salomonsen et al., 2014; Shiina et al., 2007). All *BG* genes are apparently polymorphic. How they contribute to immunity remains to be revealed. Presently only *BG1* provides a link with immune responses (Goto et al., 2009). Some BG proteins may participate in interactions with the cytoskeleton in the epithelial brush border (Bikle et al., 1996).

All BG molecules identified so far contain three structural elements. These are a single extracellular immunoglobulin-like domain of the V-region type bearing an “extra” cysteine for intermolecular disulfide linkage, a Type 1 transmembrane domain and a cytoplasmic tail composed of small domains (usually seven amino acids) predicted to form α-helical structures that pair into coiled coils (Kaufman et al., 1990; Miller et al., 1991). Diverse BG family members encoded by different loci and in distinct haplotypes maintain the three structural features but vary conspicuously in amino acid sequence and in the length of cytoplasmic tail even among isoforms encoded by the same locus (Hosomichi et al., 2008).

Specificity is apparent in the expression pattern of individual *BG* family members (Miller et al., 1991; Miller et al., 1990; Salomonsen et al., 2014; Salomonsen et al., 1991). Some members are especially well expressed on erythrocytes, where they are recognized as alloantigens that allow the *B* system haplotypes to be distinguished serologically. BG proteins are also especially well-expressed on epithelial cells, particularly those in the duodenum, caecum, and liver (Miller et al., 1990). They are also express on many types of cells important in immunity (Salomonsen et al., 1991). RT-PCR provides evidence for expression on T cells, B cells, thrombocytes, dendritic cells and other tissues (Salomonsen et al., 2014). Cell and tissue expression appears precise with pattern of expression correlating with two different types of promoters and 5′-untranslated regions (Salomonsen et al., 2014).

**Figure 4.**
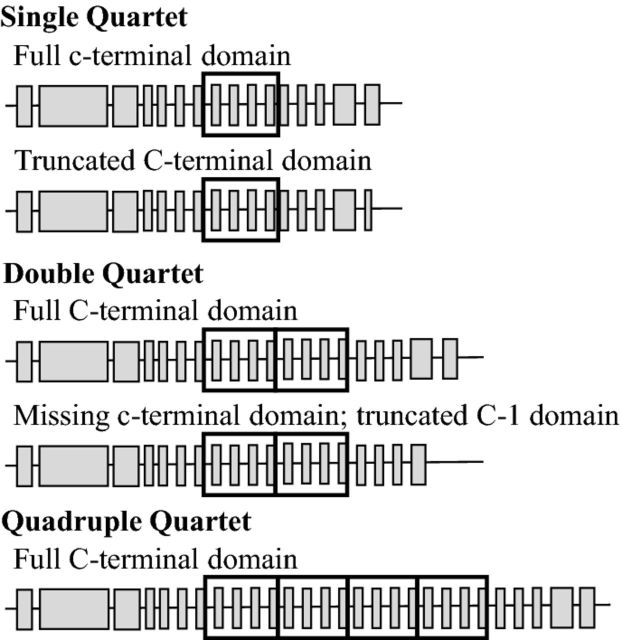
*BG1* alleles have been observed to differ in the total number of exons. This occurs through duplication/deletions of a set (quartet) of four exons. Further variation occurs at the 3′-end where exons vary in size and number so that the immunoreceptor tyrosine-based inhibition motif is present in some isoforms but absent in others. Adapted from Hosomichi et al. 2008.

*BG1*, the isolated member of the family near the class I and class II genes, is one MHC-*B* locus for which a role in disease resistance has been defined (see Marek's disease section below). BG1 isoforms encoded within different standard MHC-*B* haplotypes display three types of variability. In addition to sequence polymorphisms, alleles differ structurally in the number of the exons encoding the BG cytoplasmic heptad repeats. *BG1* alleles variously carry one, two and four copies of a quartet of exons and their associated introns (Figure 4). The significance of the variation in the coiled-coil region is not known. *BG1* alleles also vary in the number and size of exons encoding the c-terminal domain, which is apparently present only in BG1 molecules (Figure 4). As a result an immunoreceptor tyrosine-based inhibition (ITIM) motif is present in some, but not all BG1 isoforms (Goto et al., 2009; Hosomichi et al., 2008).

### TRIM/Blec Sub-Region

There are 25 genes within the *TRIM/Blec* sub-region in addition to *BG1*, already described above. The nature of these genes is summarized in Table 1. Fourteen of the 25 are assessed as “expressed genes” because their sequences match cDNA and EST sequences (Shiina et al., 2007). Seven additional genes show structural integrity, have mRNA sequence accession numbers), but are not well characterized as to ORF. Nearly all the genes in this region are thought to contribute in some way in immune defense based on homology to counterparts in other species. Their functions are thought to be in innate immunity with the *TRIM* genes providing defense against viral infections. Among the *Blec* genes, *Blec2* is considered likely to be a natural killer cell receptor perhaps working in concert with ligands encoded in the nearly MHC-*B* class I genes. Interestingly, the *Blec2* locus, also named *B-NK*, is closely related to mammalian lectin-like natural killer cell receptors (Kaufman et al., 1999; Rogers et al., 2005; Rogers and Kaufman, 2008). *Blec2* displays a relatively high level of diversity, bears a functional inhibitory motif and is well expressed in lymphoid tissues (Rogers et al., 2005; Rogers and Kaufman, 2008). In a comparison of sequences among 14 MHC-*B* haplotypes, *Blec2* has a nonsynonymous (dN)/synonymous (dS) substitution ratio of 7.0698 for SNPs within amino acid coding sequences (Hosomichi et al., 2008). This is significantly different from the expectation of sequence diversity occurring by chance (dN/dS = 1), suggesting *Blec2* is under positive selection. In contrast, *Blec1* is less polymorphic, homologous to activation-induced lymphocyte receptors, found to be well expressed only in cecal tonsils, and is quickly up-regulated following cell activation (Rogers et al., 2005; Rogers and Kaufman, 2008).

**Table 1.** *TRIM/Blec* sub-region genes within the MHC-*B* region. The genes are classified as: *expressed* if it is transcribed to mRNA and also has a reliable open reading frame and/or a known protein product; *candidate* if it is transcribed to mRNA (these have mRNA sequence accession numbers), but for which an ORF is uncertain or unknown, and *pseudogene* if partial gene sequences are present. Adapted from Shiina et al. 2007.

Gene type	Gene copies	Names	Status	Conserved Domains/motifs	Immune Defense	Known or imputed function
Tripartite motif	7	TRIM7.1, TRIM7.2, TRIM27.1, TRIM27.2, TRIM 39.1, TRIM39.2, TRIM41	Expressed/ Candidate	RING finger, B-box, coiled coil motifs, and PRY-SPRY (B30.2)	Likely	Possibly contributing resistance to pathogens
C-type lectin-like	4	Blec1, Blec2, Blec3, Blec4 (ps)	Expressed/ Candidate/ Pseudogene	C-type lectin-like genes similar to those guiding NK cell responses	Likely	Likely candidates for affecting early immune responses
Zinc finger protein	3	Bzfp1, Bzfp2, Bzfp3	Expressed/Candidate	Zinc finger protein		Likely interaction modules binding nucleic acids, DNA, RNA, proteins, or other small molecules,
transfer RNA	4	tRNA-Lys.1, tRNA-Lys.2, tRNA-Val, tRNA-Leu	tRNA	RNA cloverleaf structure		An RNA adaptor molecule physically linking the sequence of nucleic acids and the amino acid sequence of proteins
B30.2	2	B30.1and B30.2; B30.2-1 B30.2-2	Expressed/Candidate	PRY-SPRY (B30.2)	Likely	Likely helping to regulate innate and adaptive immunity
Kinesin	1	KIFC1 (Kinesin Family Member C1)	Expressed	C-terminal motor, super-helical stalk, N-terminal tail		Candidate for sliding/cross-linking microtubules
44G24	1	44G24	Candidate	Possibly DAXX		Function unknown. DAXX domain is reported to be associated with apoptosis
L-amino acid oxidase	1	LAO	Expressed	LAAO	Perhaps	A flavoenzyme which catalyzes oxidative deamination of L-amino acids and could contribute in immunity by inducing apoptosis
HEP21	1	HEP21	Expressed	uPAR/CD59/Ly-6/ snake neurotoxin superfamily	Likely	Predominantly expressed in oviduct
Guanine nucleotide binding	1	GNB2L	Expressed	G protein	Perhaps	A G proteins transduces signals from surface receptors; may help control lymphocyte activation and proliferation
BG	1	BG1	Expressed	IgV-like, transmembrane coiled-coil, ITIM motif	Likely	Likely cell activation/inhibition

### MHC Class I and Class II Gene Sub-Region

This core of the chicken MHC-*B* region contains genes involved in classical peptide antigen presentation. Within this sub-region are two MHC class I genes, two class IIβ genes for antigen presentation and genes for processing and loading antigen. Excellent early work greatly advanced the understanding of the chicken MHC-*B* class I and class II genes (Behar et al., 1988; Bourlet et al., 1988; Frangoulis et al., 1999; Guillemot et al., 1988; Guillemot et al., 1986; Kroemer et al., 1990; Zoorob et al., 1990; Zoorob et al., 1993). These studies provided both the basis for the first reported genomic sequence (*B12*) (Kaufman et al., 1999) as well as a bridge that sped the recognition of the MHC-*Y* gene region's complexity (Miller et al., 1994; Miller et al., 1996).

Both MHC class I genes, *BF1* and *BF2* encode classical MHC class I molecules (Kaufman et al., 1999; Koch et al., 2007; Sherman et al., 2008). Although fundamentally similar, the two loci are apparently functionally specialized. *BF2* serves in antigen presentation whereas *BF1* has apparently primarily another function. Several studies provide evidence that less *BF1* mRNA is typically present in cells compared to *BF2* mRNA (Livant et al., 2001; O'Neill et al., 2009; Shaw et al., 2007; Wallny et al., 2006). While all studies report that *BF1* is less well expressed, the degree to which lower expression occurs may depend upon the tissue examined. Some ratios reported are in the range of ∼1:1.5 to 1:6 (Juul-Madsen et al., 2000a; O'Neill et al., 2009), while others are greater (Juul-Madsen et al., 2000a; Shaw et al., 2007). The significance of these differences in the level of expression is unknown. Promoter sequences differ between *BF1* and *BF2.* Various deletions, insertions and rearrangements result in sequence divergence that likely affects the efficiency of expression and in some instances, the production of pseudogenes (Shaw et al., 2007). In addition, among the BF1 sequences themselves, two strongly separated lineages with differences in Enhancer A and promoter region sequences have been identified (O'Neill et al., 2009). These are interpreted as evidence that *BF1* genes are under selection for variation.

*BF1* encoded molecules may be ligands in NK cell interactions. A careful study of *BF1* allele sequences (Ewald and Livant, 2004; Livant et al., 2004) revealed a conserved locus-specific motif that resembles the motif on human MHC HLA-*C* molecules recognized in natural killer cell interactions. This motif is conserved among *BF1* alleles. In contrast, the corresponding region in *BF2* alleles is highly variable. Ewald and Livant (2004) hypothesize that BF1 molecules serve primarily in innate immunity. In favor of this hypothesis is suppression of natural cell killing of target cells displaying BF1 on their cell surface compared to controls (Zhang et al., 2012b).

Considerable progress was made in defining the structure of the *BF2* gene and demonstrating its function prior to the realization that this was the single gene in chickens for classical peptide antigen presentation (Fulton et al., 1995; Hunt et al., 1994; Kaufman et al., 1992; Kroemer et al., 1990; Pharr et al., 1994; Thacker et al., 1995). More recently, crystal structures and sequences of eluted peptides provide insights into the specificity of peptide binding in BF2 (Chappell et al., 2015; Koch et al., 2007; Sherman et al., 2008; Wallny et al., 2006). Because BF2 is the only classical class I isoform for presenting peptide antigens in chickens, the chicken provides a useful experimental model for investigating the specificity of antigen processing, binding and presentation and subsequent immune responses. A good example for this is the evidence that *BF2* is the source of protective immunity against the Rous sarcoma virus challenge (defined as tumor regression over a time course in which an adaptive immune response likely occurs) (Hofmann et al., 2003). Immunization with a single v-*src* peptide predicted to bind in the BF2*12 binding groove was sufficient to induce a protective immune response. Findings with a vaccine for infectious bursal disease provide similar evidence between the effectiveness of a molecularly defined vaccine and BF2 binding preference (Butter et al., 2013). Further worked has defined CD8+ T-cell epitopes for avian influenza (Hou et al., 2012; Reemers et al., 2012). Alternative forms of transcripts may contribute further specificity to *BF2* (and *BF1*) function (Dalgaard et al., 2003; Dalgaard et al., 2005).

In general, the same molecular interactions that occur in the peptide binding within other classical MHC class I molecules occur in the binding of peptides in the BF2 binding groove. Peptides are tethered through interactions between atoms in the peptide backbone at the amino and carboxyl termini and highly conserved residues at the margins of the ligand-binding groove. These features are confirmed in structural determinations for several BF2 isoforms (Chappell et al., 2015; Koch et al., 2007; Zhang et al., 2012a). As with other classical MHC class I molecules that bind peptide, the exact peptides bound are determined through preferential interactions with residues lining binding pockets arrayed along the length of the BF2 binding groove. Typically numerous different peptides are bound and presented in MHC class I molecules at the surfaces of cells. Although many of these are self peptides that elicit no response because of prior deletion of reactive T cells during development, these various peptides represent the bulk of peptides that elute from the MHC class I binding groove in tests to determine the peptide binding preferences of individual MHC class I molecules.

Peptide binding preferences are now available for BF2 isoforms. Motifs have been derived from sequencing of pooled peptides and from mass spectrometry (ms) identifications of individual peptides (Chappell et al., 2015; Sherman et al., 2008; Wallny et al., 2006). With the ends of the peptide bound to the margins of the groove through the main chain as is typical for MHC class I peptide binding and longer peptides assumed to be arching out of the groove (a structural study confirms this is correct (Chappell et al., 2015)) the binding preferences B2*2101 (based primarily on hydrophobic residues) were readily evident among 34 eluted endogenous peptides that varied in length from 8–12 residues. Motif scanning (http://www.hiv.lanl.gov/content/immunology/motif_scan/motif_scan) revealed that virtually all proteins produced by avian viral pathogens (other pathogens not examined) contain at least one BF2*2101 motif. Sherman and colleagues proposed that BF2*2101 is common in chicken populations because of its general utility (Sherman et al., 2008). It will be interesting to learn whether the general utility of antigen presentation in BF2*2101 is related to the constrained evolution of MDV pathogenicity observed in MHC-*B21* chickens (Hunt and Dunn, 2013).

The 30 individual endogenous peptides eluted from BF2*1301 defined a quite different binding preference (Sherman et al., 2008). Most of the eluted peptides contained eight or nine amino acids, suggesting that these bind essentially in a fully extended confirmation, recently confirmed by crystallography of the BF2*0401 isoform, which is identical to BF2*1301 in binding groove sequence (Chappell et al., 2015). This motif is based mostly on acidic residues. When the BF2*1301 motif was evaluated by motif scanning, only a few proteins from a very limited array of viruses contained the BF2*1301 motif suggesting a selective advantage for this allele only in infections with a subset of pathogens (Sherman et al., 2008). The description of BF2*2101 and BF2*1301/BF2*0401 as “generalist” and “specialist”, respectively, in a recent structural study (Chappell et al., 2015) supports the initial observations of BF2 specialization by Sherman and colleagues and extends the characterization to levels of expression at the cell surface.

Also within the MHC class I and class II gene sub-region are two *TAP* (transporters associated with antigen processing) genes, which encode ATP-binding-cassette transporter molecules that deliver peptides from cell cytoplasm into the endoplasmic reticulum (ER) where they are loaded of MHC class I molecules. Interestingly, a MDV MHC class I immune evasion gene was recently shown to target TAP (Hearn et al., 2015). Also present is *TAPBP* encoding TAP-associated glycoprotein (also known as TAP-binding protein or tapasin), a protein that mediates interaction of MHC class I molecules with the TAP molecules and facilitates peptide antigen loading. Kaufman has postulated that *TAP* and *TAPBP* alleles have co-evolved with the adjacent *BF2* alleles to optimize BF2 antigen presentation (Kaufman et al., 1999; Kaufman et al., 1995b; van Hateren et al., 2013; Walker et al., 2011; Walker et al., 2005).

MHC-*B* haplotypes have two class IIβ genes that can be described as major and minor forms (Jacob et al., 2000; Pharr et al., 1998; Pharr et al., 1993). These genes lie on either side of the tapasin gene. The major gene, always a member of the BLβII family, is always located between tapasin and RING3. The weakly expressed minor gene, located between *Blec1* and tapasin, belongs to either the BLβII or the BLβVI family (Jacob et al., 2000; Zoorob et al., 1993). A third MHC-*B* class IIβ gene appears in some haplotypes, but has not been located (Jacob et al., 2000).

Molecules that assist in MHC class II loading, the DM molecules are class II molecules that remain within intracellular vesicles and have a central role in the loading of MHC-B class II molecules with peptide. The *DM*, *TAP*, and *TAPBP* genes are retained over evolutionary time, presumably co-evolving in their roles in peptide antigen presentation with the nearby MHC class I and class II genes. The remaining gene within this region is *BRD2 (RING3)*, a chromatin binding protein that apparently has no direct role in immunity.

### Complement Gene Sub-Region

This sub-region, often referred to as the class III region of the MHC, contains five genes in chickens. Four conserved genes, C4 (complement factor C4 with a role in inflammation), CenpA (centromere protein), CYP21 (steroid 21-hydroxylase) and TNXB (tenascin XB), are present in a highly conserved order in this sub-region. The fifth gene is *LTB4R1* and likely encoding a leukotriene receptor with a role in inflammation. It is not located within or near the MHC in mammals, but has been located within the MHC of at least two avian species (Chen et al., 2015; Shiina et al., 2007).

### The CD1 Gene Sub-Region

In contrast to mammals the CD1 genes in chickens are located on the same chromosome as the MHC. The two chicken CD1 genes, *CD1-1* and *CD1-2*, are located a short distance away from the two classical MHC-*B* class I genes (Maruoka et al., 2005; Miller et al., 2005; Salomonsen et al., 2005). CD1 molecules are well studied in mammals and are known to contribute in immune responses against bacteria. Human CD1 molecules present lipids and glycolipids to specialized subsets of T cells that bear T cell receptors with remarkable specificity for the structural details of potential antigens including the capacity to distinguish self and non-self lipid antigens.

Not nearly as much is known about the two chicken CD1 molecules, although both structures have been resolved. The structures confirmed their relatedness to other members of this ancient gene family and revealed distinctive binding pockets not present in other CD1 molecules. The highly unique CD1-2 binding pocket is considered primordial because the binding groove is very small, actually more of a pore, and considered likely able to only accommodate fatty acids, or single alkyl chain lipids with up to 16 carbons (Zajonc et al., 2008). The *CD1-1* gene encodes a molecule with a larger binding region of dual-pocket, dual-cleft structural design (Dvir et al., 2010). This molecule is more similar to mammalian CD1 in its capacity to bind large dual-chain lipids.

## MHC POLYMORPHISM AND RESPONSES TO INFECTION

### MHC-*Y* in Responses to Infection

The contribution of MHC-*Y* polymorphism in differential responses to infection is not well understood. Relatively few disease challenge trials have been done looking at how MHC-*Y* polymorphism might affect the incidence of disease. To date, most experiments that have been done have asked about whether MHC-*Y* contributes in responses against MD. No major influences have been found. In a Marek's virus challenge trial using progeny from fully pedigreed families in which three MHC-*Y* and two MHC-*B* haplotypes were segregating, birds homozygous for one MHC-*Y* haplotype were at a 2.3 times greater risk for developing tumors compared with all other MHC-*Y* genotypes combined (*P* < 0.02). In other experiments with progeny from two sets of paired lines displaying genetic differences in resistance to MD, no statistical significant differences noted between the MHC-*Y* haplotypes. However, these studies noted that birds with some MHC-*Y* genotypes tended to have greater longevity within the time course of the trial, lower viremia, and fewer tumors than others (Bacon et al., 1996; Pharr et al., 1996; Vallejo et al., 1997). A fourth trial found no association (Lakshmanan and Lamont, 1998).

The influence of MHC-*Y* haplotype on the regression of Rous sarcoma virus-induced tumors has also been examined. Since some MHC-*B* region haplotypes strongly influence RSV tumor growth (see section below), tests were designed in which the MHC-*B* influence was modest. In one test with *B*2*B*5 chickens, the MHC-*Y* genotype had a significant effect on the pattern of tumor growth, tumor fate and mortality rates (LePage et al., 2000). In another test carried out with birds homozygous for *B19* that were selected over 18 generations for their capacity to regress or progress RSV tumors, MHC-*Y* haplotype was again found to have a significant, but here a more moderate effect on tumor growth (Pinard-van der Laan et al., 2004; Praharaj et al., 2004).

With the additional genome-wide assays that can be used in disease challenge trials, more data will be available defining the relationship between MHC-*Y* genes and immunity in chickens. It could be that antigen presentation by the MHC-*Y* class I genes has a primarily role in another arm of immune defense against non-viral pathogens. MHC-*Y* class I genes have been found to be up-regulated in several gene profiling studies examining responses to inoculation with bacteria and sheep red blood cells (Connell et al., 2012; Geng et al., 2015; Wu et al., 2015).

### MHC-B in Responses to Infection and Vaccination

Early findings that the major MHC-*B* haplotypes influence the MD incidence had a significant impact on research focused on the chicken MHC. Investigations have concentrated both on the mechanisms underlying this major MHC-*B* haplotype influence on MD and on learning whether MHC-*B* genetics affect immune responses to other diseases. The literature is now extensive. MHC-*B* has been examined for a role in resistance to a wide variety of diseases caused by viral, bacterial and parasitic pathogens.

Investigations into disease responses have been carried out in various ways. Some compare responses in inbred congenic and sometimes MHC-*B* recombinant lines. Others examine populations in which MHC-*B* types are segregating either in selected lines or, occasionally in natural or semi-natural populations. Here we attempt to provide a very brief overview of investigations into MD as well as diseases caused by Rous sarcoma virus (RSV), v-*src* DNA, other viruses, several bacterial infections, coccidiosis, and an infestation with avian ectoparasites. More information is available for the role of MHC-*B* in MD and RSV than in other diseases.

### Marek's Disease (MD) Virus Infections and Vaccinations

The first association with MHC-*B* reported appeared in an abstract (Hansen et al., 1967). The outcome of a MD virus (JM Strain) challenge trial of fully pedigreed backcross families in which two MHC-*B* (*B21* and *B19*) haplotypes were segregating clearly showed the major role of the MHC-*B* in resistance to Marek's disease (Briles et al., 1977). This study showed an eightfold difference in MD tumor incidence between MHC-*B* haplotype and genetic resistance to MD. Another study published the same year provided evidence for a role of MHC haplotype in active restriction of transplanted MD tumor growth (Longenecker et al., 1977). Further work helped to rank the contributions of additional MHC-*B* haplotypes in congenic lines and to reveal how MDV virulence affects MHC-*B* encoded resistance (Abplanalp et al., 1984). There is further evidence for the contribution of additional MHC-*B* haplotypes in MD (Bacon et al., 1983; Bacon et al., 1981). MHC-*B* haplotype also influences the efficacy of vaccination against MDV (Bacon and Witter, 1992, 1993, 1994a; Bacon and Witter, 1994b; Bacon and Witter, 1994c, 1995). Additional studies show that non-MHC-*B* genes also contribute in responses again MDV (Bacon et al., 2000).

Advances in identifying the individual genes within MHC-*B* contributing to the MHC-*B* influence on MD were made through the success of Elwood and Ruth Briles in identifying recombinant haplotypes (Briles and Briles, 1980). In early work, MHC-*B* linked resistance to MD was assigned to “a gene or genes, within or closely linked to the B-F region of the B complex” with one of several recombinant haplotypes isolated for this purpose (Briles et al., 1983). It is important to note here that the responsible gene was not identified. When genomic sequence for MHC-*B* became available it was possible to localize the crossover breakpoint just upstream of the *TRIM7.2* gene (see location of *TRIM7.2* in Figure 3) leaving all genes downstream from the breakpoint as candidates for contributing in MHC-*B*–linked resistance to MD (Shiina et al., 2007).

Further advances were made with two additional MHC-*B* recombinant haplotypes, *BR2* and *BR4*, identified as “identical” by the serological markers then available (Briles and Briles, 1980; Plachy et al., 1984). Realizing that chromosomal recombination breakpoints rarely occur at precisely the same location, a small study tested these two recombinants in developing congenic lines (003.R2 and 003.R4) at the fourth backcross to determine if they had identical phenotypes with respect to MD resistance (Schat et al., 1994)**.** The evidence found indicated that 003.R2 and 003.R4 chickens differed in resistance to MD. In a follow-up study, 003.R2 and 003.R4 lines were tested again with larger numbers of animals after 10 backcross generations. Highly significant differences in MD mortality and total MD incidence were found between the 003.R2 and 003.R4 lines (Goto et al., 2009). The 003.R2 birds exhibited 10% mortality during trial and 19% MD overall, while the 003.R4 birds showed 39% mortality during trial and 47% MD incidence. This study mapped the crossover breakpoints in the *BR2* and *BR4* haplotypes to different positions within the *BG1* locus showing that the BG1*R4 and BG1*R2 alleles were completely identical in coding region sequence, but differed in the 3′-untranslated region (3′-UTR). In the *BR4* 3′-UTR a 225 bp insert of retroviral origin was present. The 3′-UTR difference had little influence on transcription but produced a consistent difference in assays for translation. The consequence of this modification is not yet fully understood, but it may place BG1*R4 transcripts under post-transcriptional control that results in altered BG1 activity. Given the presence of the ITIM motif (associated with attenuation of lymphoid cell activation) in both isoforms it could be that the difference in the MD incidence is related to BG1 signaling differences as the result of repression of BG1 translation encoded by the *BR4* haplotype. This study provides strong evidence that at least part of the long observed genetic influence of MHC-*B* in MD can be attributed to *BG1* and reveals another way in which the MHC-*B* region contributes to disease resistance in addition to specific antigen presentation.

### Inoculation with Rous Sarcoma Virus (RSV) and v-*src*

Tumors induced by Rous sarcoma virus (RSV) are mediated by the oncogene v-*src* (Brugge and Erikson, 1977). A critical MHC-*B* influence on the fate of Rous sarcomas was first reported in two concurrent publications (Collins et al., 1977; Schierman et al., 1977). Inbred line crosses G-B1 (*B13B13*) and G-B2 (*B*6*B*6) were tested for their response against Rous sarcomas. Backcross progeny from the [G-B1 x G-B2] x G-B1 mating showed differential tumor growth with 94% tumor regression in the heterozygous *B6B13* birds compared with 4% tumor regression in birds homozygous for *B13* (Schierman et al., 1977). A simultaneous study with the F_2_ generation of inbred lines 6_1_ (*B*2*B*2) x 15_1_ (*B*5*B*5) showed differences in tumor regression among individuals in a challenge experiment. Tumor regression scores were 76% for *B*2*B*2, 35% for *B*2*B*5, and 0% for *B*5*B*5. In addition, metastasis, tumor spread to distant sites, was higher in genotype *B*5*B*5 than in *B*2*B*2 (Collins et al., 1977). A third study with two additional MHC-*B* haplotypes (*B12* in Line CB and *B4* in Line CC) in Prague, also confirmed a major MHC-*B* role in the regression of tumors induced by v-*src* (Plachy and Benda, 1981). Further studies with MHC-*B* recombinant haplotypes have been conducted to localize the gene or genes within MHC-*B* that determine Rous sarcoma tumor regression. Studies comparing the recombinant haplotype *BG4-BF12* (in Line CB.R1) mapped resistance to the portion of MHC-*B* identified by BF alloantigens (Plachy and Benda, 1981). The opposite outcome, tumor progression, was found with two additional recombinant haplotypes, *CC.R1* and *CC.R2*, in which the *BF* sub-region originated from *B4* and the *BG* sub-region from *B12* (*BG12-BF4*) (Plachy et al., 1984). Further studies with congenic recombinant lines produced on inbred Line UCD 003 background again mapped the MHC-*B* influence in RSV tumors to the sub-region identified by BF alloantigens. Line 003.*R2* (*BG*23-*BF*2) and Line 003.*R6* (*BG23-BF21*) had lower tumor scores compared with four other recombinants (White et al., 1994).

A hint for additional complexity of the MHC-*B* region role in disease resistance was seen in RSV tumor regression trials with MHC-*B BR2* and *BR4* recombinant haplotypes. Lines 003.R2 and 003.R4, as occurred with MD, have been reported to respond differently to RSV tumors (Schulten et al., 2009), suggesting a contribution from *BG1*. Other genes also influence the outcome of RSV infections. Tumor growth differed among MHC identical lines with dissimilar background genomes, which indicated a role for non-MHC genes. Furthermore, RSV tumor metastases differed according to non-MHC background as well as MHC-*B* genes (Taylor, 2004).

A direct response to v-*src* protein was supported in further studies. Injection of RSV v*-src* DNA produced tumors whose growth and outcome depended on chicken line (Halpern et al., 1991). Those tumors had no viral replication or viral structural sequence. MHC control of v-*src*-induced sarcomas was demonstrated in congenic lines 6.6-2 (*B*2*B*2), a regressing line and 6.15-5 (*B*5*B*5), a progressing line (Taylor et al., 1992), consistent with earlier findings with RSV (Collins et al., 1977). For v-*src* tumor metastases, the *B*2*B*2 line (6.6-2) had lower metastases than the *B*5*B*5 line (6.15-5) (Taylor et al., 1994). Lines 6.6-2 also had greater immunity to a second v-*src* challenge. The response was very likely directed against some antigen determined by v-*src* (Taylor et al., 1994; Taylor et al., 1992); perhaps the v-*src* C-tail antigen (Hofmann et al., 2003). Similar results were shown in the Prague congenic lines as v-*src* tumors regressed in Lines CB (*B12B12*), and CB.R1 (*BG4-BF12*) regressed v-*src* tumors compared with Line CC (*B4B4*) in which tumors progressed (Svoboda et al., 1992). Line CB had higher immunity against a second challenge of v-*src* or RSV following primary v-*src* injection compared with Line CC (Plachy et al., 1994). Clearly MHC-*B* makes a significant contribution to the immune responses underlying the regression of RSV induced tumors. BF2 molecules may contribute specificity in the presentation of v-*src* protein (Hofmann et al., 2003). Perhaps BG1 molecules affect cell responsiveness to initial infection.

### Susceptibility to avian leukosis virus (ALV)

ALV infections are also affected by the MHC*-B* haplotype. In the F_3_ or F_4_ generation of inbred line crosses (6_3_ × 15_1_) producing *B*2*B*2, *B*2*B*5 and *B*5*B*5 progeny, *B*2 haplotype was associated with lymphoid leukosis resistance as well as resistance to MD and the ability to regress RSV tumors. Haplotype *B*5 was found generally associated with virus susceptibility indicating a general deficiency in anti-viral disease response (Bacon et al., 1981). In a second study including *B*2, *B*5 from another source (15I_4_) and *B15*, *B5 and B15* were found to be highly and essentially equally susceptible to tumors induced by MDV, RSV, and ALV (Bacon et al., 1983). This finding further highlights the resistance conferred by *B2* haplotype.

The MHC-*B* region was examined in an Australorp population for an influence in avian leukosis virus (ALV) group-specific (**gs**) antigen shedding related to virus infection. Haplotype frequencies for three MHC-*B* haplotypes were B8a = 66.7%, *B9a* = 15.6, and *B21* = 17.8% in a base population. The data supported differential susceptibility to horizontal virus transmission based on MHC-*B* complex type but no MHC-*B* effect on vertical transmission (Yoo and Sheldon, 1992).

### Responses to Newcastle Disease (ND) Virus

The role of MHC type has also been investigated in studies of immune responses to Newcastle disease virus (NDV), an avian paramyxovirus that causes respiratory disease that varies from extremely pathogenic to mild and unnoticed depending on strain. When tested with a live attenuated ND vaccine lines birds bearing different MHC types were found to vary in specific T cell responses (Norup et al., 2011). Significantly greater proliferation of CD4+ T cells was observed in birds of *B13* haplotype compared to *B130* haplotype in NDV antigen-specific recall responses (evidence of difference in adaptive immunity). MHC haplotypes also contribute in the antibody titers produced by immunization with NDV (Dunnington et al., 1992). A lower anti-NDV antibody titer was observed in *B21B21* chickens compared to *B13B13* and *B13B21* birds in the white leghorn populations selected for high and low antibody responses to sheep red blood cell antigens. This observation provides additional evidence that MHC haplotype differences affect adaptive immune responses.

### Responses to infectious bursal disease (IBD) Virus

IBD is highly contagious in young chickens. IBD virus targets are lymphoid tissues, especially the bursa. In an investigation of antibody responses to a live attenuated commercial IBDV vaccine among three different lines in which four different MHC-*B* haplotypes were segregating, birds with one haplotype (*BW1*) were found to have significantly higher serum antibody titers than birds with the three other haplotypes (*B19*, *B21* and *B131*) (Juul-Madsen et al., 2002). *BW1* was also associated with significant lower bursa lesion scores. In another study by the same research group with killed IBDV vaccine, MHC also affected antibody response (measured by ELISA) in additional lines (Juul-Madsen et al., 2006). Birds with some MHC haplotypes were low responders (*B19*, *B12* and *BW3*) and others high responders (*B201*, *B4*, and *BR5*). Further work in this study highlights how various factors, such as various regions within the MHC-*B* region, age, and background genes, influence specific antibody responses. These studies provide another example of how adaptive immune responses are affected by the MHC genotype.

### Infection with infectious bronchitis (IB) Virus

IBV, a coronavirus, causes a highly contagious, acute respiratory disease in chickens. The MHC basis for an earlier observation of a dominant autosomal resistance gene for IB (Bumstead et al., 1989) only recently became clear in a retrospective study of vaccination of young birds from MHC congenic strains (Bacon et al., 2004). These findings were supported by an additional study with more MHC-*B* haplotypes that also ranks the haplotypes and demonstrates dominance of the resistance gene (Banat et al., 2013). The contribution of MHC-*B* to IB is particularly intriguing because the rapid progression of disease following infection. Dramatic differences between MHC-*B* haplotypes in the innate immune response of macrophage maturation has been proposed as contributing to the IBV infection differences observed among some MHC-*B* types (Dawes et al., 2014).

### MHC-B and Fowl Cholera

*Pasteurella multocida* infections cause fowl cholera in chickens. There is relatively little information available on the influence of host genetics in fowl cholera. One study using a three-marker assay for MHC-*B* (serological MHC-*B* type, humoral immune response to a amino acid polymer composed of glutamic acid, alanine and tyrosine, and RSV tumor responses) showed survival following inoculation with *P. multocida* was linked with MHC-*B* type in the Iowa State S1 line of white leghorn chickens (Lamont et al., 1987). In another study comparing indigenous and commercial breeds of chickens in Vietnam, the indigenous breed appeared more susceptible to fowl cholera than the commercial breed, with the susceptibility apparently linked to a select set of MHC haplotypes defined by LEI0258 (Schou et al., 2010).

### Influence of MHC-B in Staphylococcal Infections

Two substantial studies provide evidence for MHC-*B* haplotype influencing the outcome of infections with *Staphylococcus aureus*. Comparisons with 10 UC Davis inbred congenic leghorn lines showed significant differences in mortality among chicks challenged at 3 d of age with a moderately pathogenic isolate and a highly pathogenic isolated of *S. aureus* (Cotter et al., 1992). There was lower mortality in the Line 331 (*B2/B2*) chicks compared to chicks in Line 336 (*BQBQ*), Line 335 (*B19B19*) and Line 330 (B21/B21). No significant difference was found among the lines in similar trials with birds at six wk of age. Another group studying bacterial skeletal disease in a genetically pure line of broiler breeder chickens observed highly significant association of two MHC-*B* homozygous genotypes (*BA4BA4* and *BA12BA12*) with lameness in which *Staphylococcus* spp. was isolated from the skeletal lesions (Joiner et al., 2005).

### MHC-B and Salmonella Species

Because *Salmonella* spp. are a major cause of foodborne illness in man, and poultry products are a major source, there is considerable interest in finding ways to reduce the incidence of *Salmonella* infections in flocks raised for food. Although most *Salmonella* serovars produce little disease in adult chickens, these bacteria often colonize the intestinal tracts of poultry. Immunity is considered a primary means of controlling colonization and hence there is an interest in immunogenetic links with infection. A study with inbred congenic lines showed that at as early as 3 d of age resistance to *Salmonella* is expressed (Cotter et al., 1998). Different MHC-*B* haplotypes contribute differently to resistance ((high in Line 342 (BCBC); lower in Line 253 (B18B18) and Line 254 (B15/B15). Using microsatellite markers for mapping, MHC class I was identified as a gene associated with *Salmonella* colonization (Lamont et al., 2002). Further work linked resistance to the MHC I and class II via a sequence polymorphism and SSCP (Liu et al., 2002), and to antibody response kinetics (Zhou and Lamont, 2003). The indigenous and commercial breeds of chickens in Vietnam also linked *Salmonella*-specific antibody responses to MHC-*B* haplotype (Schou et al., 2010). A QTL study of a (N × 6_1_) × N backcross population identified MHC-*B* as making a small contribution to the *Salmonella* carrier-state (Tilquin et al., 2005). Differences in responses between birds with different MHC-*B* haplotypes may become less with increasing age (Barrow et al., 2004; Cotter et al., 1998).

### MHC-B and Escherichia coli Infections

In a study comparing *B21* and *B13* haplotypes in birds 4 wk of age, MHC-*B* haplotype was implicated in the likelihood of an individual bird developing cellulitis, an inflammatory process induced by *E. coli* that causes tissue lesions (in this experiment induced by subcutaneous injection of *E. coli*). Interestingly *B13* haplotype confers more resistance to cellulitis compared to *B21* and two additional haplotypes *BA9* and *BA12* were intermediate in incidence (Macklin et al., 2002; Macklin et al., 2009). Among those birds which did developed cellulitis, there was no link between MHC-*B* haplotype and the severity of the lesions (Macklin et al., 2002; Macklin et al., 2009). In another study by another research group, the outcome of intratracheal inoculation of adult birds with a field strain of avian pathogenic *E. coli* mortality was found to be significantly lower in *B21/B21* hens compared to *B19/B21* hens (Cavero et al., 2009).

### Coccidial Infections and MHC-B

*Eimeria* spp*. (*principally *E. tenella, E. maxima*, and *E. acervulina*) are obligate intracellular parasites that cause coccidiosis in birds. Infection produces intestinal destruction, blood loss, nutrient malabsorption, and mortality in the animals most severely affected. There is clearly a genetic influence with large differences in the coccidiosis resistance described between lines; see for example (Pinard-Van Der Laan et al., 1998; Uni et al., 1993). Additional studies have shown MHC-*B* genetic effects in coccidiosis in some genetically defined stocks (Caron et al., 1997; Clare et al., 1985; Kim et al., 2008); but not in others (Lillehoj et al., 1989; Ruff and Bacon, 1989). The outbreak of coccidiosis in a semi-natural red Jungle fowl population points to a single MHC-*B* haplotype conferring susceptibility, the contribution of which was masked by all of the six other haplotypes in heterozygous birds (Worley et al., 2010). The observation that multiple low-dose exposures to *E. tenella* stimulate immunity more effectively than a single higher-dose challenge suggests activation of adaptive immunity and that immunization occurs with time, implicating MHC-*B* in antigen presentation in the response. However, which individual genes contribute in responses to coccidial infections has not been determined.

### MHC-B and the Incidence of Intestinal Ascarid Infections

With poultry increasingly being produced under conditions with free outdoor access the likelihood of diverse infections increases including infection with parasitic worms, such as *Ascardia galli*. As with other infectious disease in chickens, there is evidence for genetic factors influencing helminth infections (Schou et al., 2003; Schou et al., 2007). A statistically significant contribution from MHC-*B* in resistance to *A. galli* has been noted in indigenous and exotic chicken in Vietnam (Schou et al., 2007). The MHC-*B* region appears to affect infection intensity (Schou et al., 2010). A study comparing two inbred lines with different MHC-*B* haplotypes revealed significantly different levels of infection and antibody titers (Norup et al., 2013). Levels of infection and antibody titers were inversely related suggesting that humoral immunity may be involved in the disease response but that higher antibody titer does not result in clearing of infection.

### MHC-B Haplotype Involvement in Resistance to Northern Fowl Mite (NFM) Colonization

Northern Fowl Mite (**NFM**; *Ornithonyssus sylviarum*) is the most common ectoparasitic arthropod in poultry production areas in the United States (Arends, 2003). MHC-*B* haplotypes appear to contribute differentially to the success NFM colonization. Trials that included UC Davis congenic lines homozygous for MHC-*B* haplotypes (UCD-254, *B15*; UCD-331, *B2*; UCD-253; *B18* and UCD-330, *B21*) and a commercial layer line (Hy-Line W-36) in which three MHC-*B* types (*B2*, *B15* and *B21*) segregate, revealed *B21* birds to be significantly more resistant to mite infection compared with *B15* birds (Owen et al., 2008). While it remains to be determined which MHC-*B* gene or genes contribute resistance to this ectoparasite, it has been suggested that this MHC-*B* encoded resistance is related to inflammatory response, with a greater degree of skin inflammation during an infestation conferring resistance by the accompanying swelling, thus limiting access of mites to blood sources in the skin (Owen et al., 2009).
